# Sport-Related Affective Benefits for Teenagers Are Getting Greater as They Approach Adulthood: A Large-Scale French Investigation

**DOI:** 10.3389/fpsyg.2021.738343

**Published:** 2021-10-11

**Authors:** Annie Carton, Alexis Barbry, Jérémy Coquart, Hervé Ovigneur, Camille Amoura, Gabor Orosz

**Affiliations:** ^1^Univ. Artois, Univ. Lille, Univ. Littoral Côte d’Opale, ULR 7369 - URePSSS - Unité de Recherche Pluridisciplinaire Sport Santé Société, Liévin, France; ^2^Centre des Transformations des Activités Physiques et Sportives, Université de Rouen-Normandie, Rouen, France; ^3^L’Institut des Rencontres de la Forme, Wattignies, France; ^4^Univ. Lille, Univ. Artois, Univ. Littoral Côte d’Opale, ULR 7369 - URePSSS - Unité de Recherche Pluridisciplinaire Sport Santé Société, Lille, France

**Keywords:** adolescents, positive affect, sports club membership, positive psychology, broaden-and-build, physical activity

## Abstract

The present investigation examined how sports club membership is related to adolescents’ daily negative and positive affects as they age. Robust prior results demonstrated that sports club membership is positively related to positive affect and negatively related to negative affect. However, surprisingly, no prior studies examined whether these benefits are consistently present throughout the teenage years or there are certain critical periods when teenagers can affectively profit more from being members of a sports club. The present cross-sectional investigation examined these questions on a comprehensive sample of French adolescents (*N*=17,337, female=7,604, aged between 10 and 18, *M*_age_=12.45years, *SD*_age_=1.94years). Besides the expected affective benefits of a sports club membership, there was no interaction between age and negative affect. However, late adolescents reported greater daily positive affective benefits of sports club membership than early adolescents. These results suggest that late adolescents can use the extra affective benefits of sports club membership to gain advantages for the first steps of their adult life, such as coping with career start or transition to higher education. These results can provide guidelines for future studies to prioritize late adolescents with heightened positive sport-related affective benefits. It can also be useful information to promote sport among late adolescents.

## Introduction

In the past 20years, robust results suggest that regular participation in physical activity and sport has a beneficial effect on physical and mental health at all ages and for a broad variety of people (Biddle and Mutrie, 2001; Haworth and Lewis, 2005; Eime et al., 2013; Gisladottir et al., 2013b). For example, Gisladottir et al. (2013b) examining more than 10,000 14-16-year-old adolescents found that practicing sports in a club is associated with a better body image (*d*=0.6), a better physical (*d*=1.06) and mental condition (*d*=0.76). These positive effects appear to be rather large. Adolescence is no exception, physical activity has a great impact on mental health in this critical and vulnerable period (Janssen and Leblanc, 2010; Ahn and Fedewa, 2011; Biddle and Asare, 2011; Rodriguez-Ayllon et al., 2019). More precisely, several authors (Richman and Shaffer, 2000; Brettschneider, 2001; Liu et al., 2015) found that doing sports is associated with a more positive self-concept and increased self-esteem, both of which are reliable indicators of mental health. Bang et al. (2020) demonstrated that belonging to sports clubs predicted positive self-esteem and also improved school engagement. Prior work reported that sport activities increased feelings of physical competence, satisfaction with physical appearance, and resulted in positive body image, which can lead to an increase in overall self-esteem (Findlay and Bowker, 2009; Zamani Sani et al., 2016; Gomez-Baya et al., 2019). Biddle et al. (2019) demonstrated that physical activity tends to decrease symptoms of depression which is among the most common mental disorders in this age group (Saxena et al., 2013). Physical activity benefited the social aspects of mental health as adolescents could develop positive social relationships through their collective practice (see Eime et al., 2013). The lack of physical activity during adolescence is correlated with poor mental health (Hume et al., 2011; Gunnel et al., 2016). All in all, it appears that physical activity and sport have a positive impact on mental health and this is especially true regarding hedonic wellbeing (maximizing positive emotions and minimizing negative ones, Diener, 2000). Indeed, sports practices allow adolescents to experience positive emotions in various ways. The broaden-and-build theoretical framework (Fredrickson, 2001) posits that positive emotions have various beneficial effects on psychological functioning and we suppose that it can provide an explanation why and how adolescents can gradually benefit from sports club membership. According to this theory, positive emotions increase physical, personal, and social resources that mobilize and unleash their potentials. Positive affects can trigger broad, curious, and optimistic thought patterns as well as spontaneous and energetic behaviors. Positive emotions accumulate over time in ways that incrementally build people’s enduring resources.

In the light of the abundance of scientific studies, it is surprising that we have very little accumulated scientific knowledge about (1) the developmental aspects of these benefits, and (2) how physical activity or sports club influences daily negative and positive affects along adolescence. The present paper aims to fill this gap among adolescents aged between 10 and 18 and explore the potential affective benefits of belonging to a sports club throughout adolescence.

During and after physical activities, people experience various affective benefits that can contribute to their wellbeing. One of these is related to physical feeling states that capture sensory experiences (e.g., arousal). These experiences are distinct from the general positive/negative affects that might appear as the benefit of physical activities (Dunton et al., 2014). These two aspects have not the same duration, level of intensity and even the underlying cognitive processes are different. In the present study, instead of the sensory experiences, we will focus on core affects (Russell, 1980; Russell and Barrett, 1999); the most elementary, consciously accessible emotions and we will distinguish them based on their positive or negative valence. In the present case, positive affects included states, such as joy, enthusiasm, pride, and full-of-energy, while negative affects included anxiety, fear, sadness, shame, and guilt (Van Geel and De Mey, 2003). Our main goal was not assessing affects during or right after physical activities, but we aimed to explore these affective states in a more general way, focusing on adolescents’ feelings in the few days before the assessment.

There is a consensus in the literature that sports and exercise elevate positive and reduce negative affects among not only adults, but adolescents as well (see, for example, Buckworth et al., 2013). In a recent meta-analysis, Bourke et al. (2021) demonstrated positive associations between physical activity, feeling energized, positive emotions, and vitality among children and adolescents. Their participation in sports club can also increase their social abilities (Sabiston et al., 2016; Vella et al., 2017). Being a member of a sports club has a positive influence on the adolescents’ self-esteem (Brettschneider, 2001) and social competence (Howie et al., 2010). These benefits are important during the emotionally challenging times of adolescence. Considering the dynamics of emotions in this critical period of development, it appears that positive affects decline and negative affects increase throughout adolescence (Weinstein et al., 2007; Forbes et al., 2010; Frost et al., 2015; Martin-Krumm et al., 2017). Adolescents clearly benefit affectively from physical activities (Kühnhausen et al., 2013; Cushing et al., 2018), yet it is still unclear exactly in which part of this period they can benefit the most.

### The Present Research

The present work focuses on an under-investigated topic at the intersection of developmental, positive, and sport psychology. The results will be interpreted in the positive psychological theoretical framework broaden-and-build. Namely, how sports club membership can be related to increased positive and reduced negative affects throughout adolescence. Contrasting to prior studies, the present work is particular in its relatively large sample of adolescents of various ages (ranged between 10 and 18).

Based on (1) the systematic review of Liao et al. (2015), physical activity and sports club participation (Miller and Hoffman, 2009) improve daily positive affective states; (2) robust prior findings show that adolescents experience more negative and less positive affects over time (Weinstein et al., 2007; Forbes et al., 2010; Frost et al., 2015; Martin-Krumm et al., 2017); and (3) the fact that adolescents affectively benefit from physical activities (e.g., Buckworth et al., 2013), we expect that in the later parts of adolescents will experience more affective benefits from sports club membership than the earlier parts of adolescents.

## Materials and Methods

### Procedure and Participants

This study was conducted in accordance with the Declaration of Helsinki and with the approval of the National Ethics Committee Board (n°00012476–2021-28-05-109). Participants for this study were recruited through the project of the “*French physical and mental health inventory*” program. Besides the ethical permission, the data gathering, and its further use were approved by the National Commission on Informatics and Liberty (RF 1232206). As the respondents were minors, written and signed informed consent was obtained from their parents. Respondents of the survey were informed about the content of the investigation, and they were requested to indicate their intention for participation (provide assent).

The dataset was part of an extensive, multi-year data gathering (between 2008 and 2019). For the present paper, we only focus on the data of adolescents who reported their sports club membership status. The sample consisted of 17,337 adolescents (7,604 girls, 43.86%). Most of them (12,895, 74.39%) reported that they belong to a sports club and approximately one quarter of the participants did not belong to any sports club. The age range was 10–18years among both the members of the sports club (*M*=12.45years, *SD*=1.94) and those who are not members (*M*=12.45years, *SD*=1.95), and their mean age was almost identical (*M*_all_=12.45years, *SD*_all_=1.94years). The sample was uneven regarding year groups.^1^

### Measures

#### Positive and Negative Affects

Among other measurements (height, body mass, and physical fitness level), participants were requested to describe how much they experienced a set of positive and negative emotions in the past 3 or 4days. The survey was completed 30min after a physical fitness test. Seven adjectives described negative affects: *angry, sad, anxious, ashamed, guilty, annoyed*, and *worried*; five described positive ones: *joyful, enthusiastic, proud, full-of-energy*, and *happy*. Responses were provided on a five-point Likert scale ranging from 1 (not at all) to 5 (very much).^2^

#### Sports Club Participation

Before the assessment of physical fitness level and after the measurements of height and weight, the participants responded to the following question: “Are you a member of a sports club?” Respondents could answer this question with a simple “yes” or “no.” If the answer was “yes,” they could report the sport they practiced the most often.

## Results

### Analytic Strategy

We examined the effect of sports club participation and its interaction with age (as a continuous variable) on the positive and negative affects in separate models. For this purpose, OLS regressions were conducted with using R [as well as for plotting the figure (ggplot2)]. The data met the assumption of independent errors (Negative Durbin-Watson value=1.76 and Positive Durbin-Watson value=1.90). The histogram of standardized residuals showed that in both the positive and negative affect models, the data included approximately normally distributed errors. It was also the case regarding the normal P–P plots of standardized residuals. In the positive affect plot, points at the two extremes of age were not completely on the line, but close. Finally, both the positive and negative scatterplots of standardized residuals indicated that the data met the assumptions of homogeneity of variance and linearity.

### Psychometric Properties of the Affect Measures

We supposed that the 12 items belong to two factors. Based on an exploratory factor analysis, conducted in R with varimax method as the two factors were almost independent (*r*=0.15), indicating two sharply separate factors (positive loadings ranged between 0.45 and 0.72; negative loadings range between 0.66 and 0.85, with the largest cross-loading of 0.13), a confirmatory factor analysis was conducted (in R, lavaan) with two first-ordered factors, with robust maximum likelihood estimator.

### Main Results

#### Negative Affect

**Figure 1:**
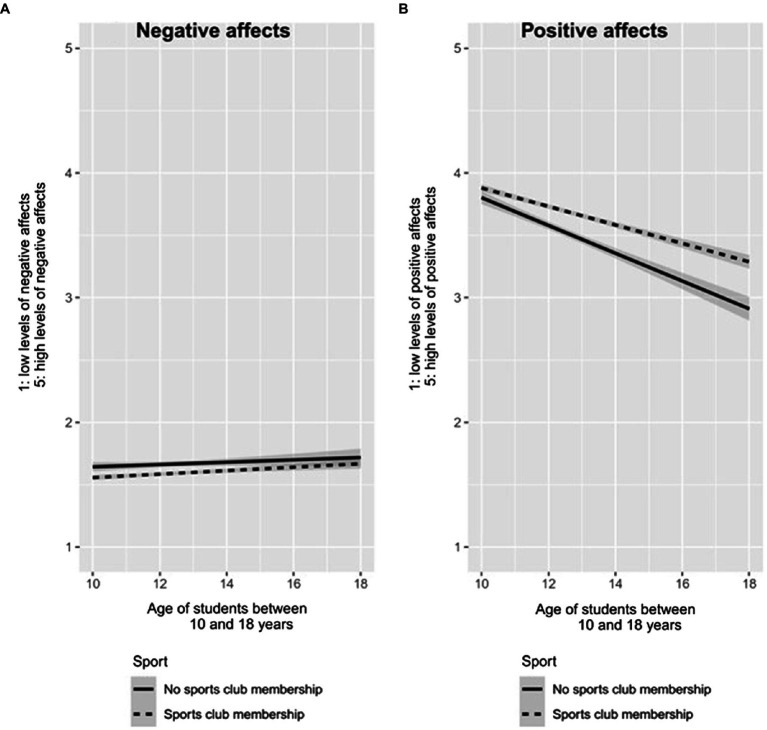
Negative and positive affects along sports club membership and age of adolescents. The left panel **(A)** depicts negative affect-related results, and it indicates a significant main effect of the sports club membership. The right panel **(B)** depicts the main effect of the sports club membership on positive affects, as well as its interaction with the age of the students showing that late adolescents experience more positive emotions than early adolescents if they belong to a sports club (compared to the lack of membership).

Adolescents who did not belong to any sports clubs experienced more negative affects then their peers belonging to a sports club, *β*=−0.09, *t*(17,337)=−4.33, 95%CI (−0.13; −0.04), *p*<0.001, *d*=0.09. However, the interaction between age and sports club participation was not significant (*p*=0.503), *R*^2^=0.003 see Figure 1A.

#### Positive Affect

Those respondents who indicated their sports club participation, experienced more positive affects in the few days before the assessment than their peers, *β*=0.20, 95%CI (0.16; 0.24), *t*(17,337)=9.83, *p*<0.001, *d*=0.20. Age also had a negative main effect on positive affect, *β*=−0.52, 95%CI (−0.60; −0.45), *t*(17,337)=−13.66, *p*<0.001, *d*=0.52. Examination of the interaction revealed that the sports club membership was associated with higher levels of positive affect among late adolescents than early adolescents (compared to those who do not belong to any sports clubs), *β*=0.18, *t*(17,337)=3.95, 95%CI (0.09; 0.26), *p*<0.001, *d*=0.18, *R*^2^=0.029, see Figure 1B. With using Fisher’s r-to-z comparison, we found that the negative relationship between age and positive affect was stronger (*z*=4.89, *p*<0.001) among students who do not belong to sports clubs (*β*=0.52, *t*=−13.40, *p*<0.001) compared to their peers who belong to sports clubs (*β*=−0.35, *t*=−15.52, *p*<0.001).

## Discussion

Being a member of a sports club during adolescence has tremendous benefits as it is associated with enhanced social competence, a better health profile, and with more positive emotions and less negative ones (Brettschneider, 2001; Howie et al., 2010; Eime et al., 2013; Gisladottir et al., 2013a). Considering these solid prior results, our main goal was to examine a missing piece in the relevant literature at what age can adolescents benefit the most emotionally from sports club membership? Potential affective benefits for late adolescents—that the present results suggest—can be especially relevant, as many career and life decisions must be made in this period. In the following, we aim to provide some explanations for this effect, discuss some nuances, highlight some limitations, and propose some questions for future studies focusing on this topic.

The positive emotional balance deriving from practicing sports can be interpreted in the light of the broaden-and-build theory of Fredrickson (1998, 2001). More precisely, according to this theory, positive emotions can accumulate and build enduring resources over time. They can also create a spiral leading to an increased likelihood of experiencing further positive emotions in the future. For example, when adolescents are experiencing positive emotions, they become more resilient in coping with potentially emerging negative feelings. Therefore, it is possible that the affective benefits deriving from sports club membership can not only serve as a source of positive emotions, but also as an antidote to negative emotions.

The framework of the broaden-and-build theory can be connected to prior sport-related results. For example, adolescents engaged in a sports club progressively develop a positive physical self-image as the result of their satisfying body-related experiences, progress in motor control, and sport-related successes (Bowker, 2006; Gomez-Baya et al., 2019). The increased physical self-image can be also linked to physical competence and can also become a key component of global self-esteem (Marsh, 1986; Sonstroem, 1997; Fox, 2000; Richman and Shaffer, 2000; Bowker, 2006; Chen et al., 2012). To put it simply, such a potential chain reaction of physical activities makes adolescents feel good about themselves. It is possible that broad-scale positive effects do not happen overnight and require years of practice. Until now, we only discussed individual characteristics and aspects; however, adolescents are fundamentally social, and these sports club-related benefits can have an effect on their functioning in their social groups, as well.

The explanation of the broaden-and-build theory can be extended to the social aspects of physical activity, such as the sports club context. Within sports clubs, among various experiences, positive affects can be socially shared and can reinforce the bonds between club members (Rimé, 2009). In a recent study, Brown and Fredrickson (2021) put emphasis on co-experiencing positive emotions. Compared to emotions experienced individually, such collective experiences can lead to greater longevity and magnitude of the positive affects. Besides these immediate effects, teens can gradually develop a network of friends in a sports club that can prevent them from feeling lonely. Findlay and Coplan (2008) suggested that sports club participation may play a unique protective role for shy individuals who have difficulties initiating contact with peers. The social context of the sports clubs might not only increase the sense of belonging (Baumeister and Leary, 1995), but also it can serve as a “training ground” for developing social competencies (e.g., Bedard et al., 2020). Moreover, it appears that along with other competencies, social skills acquired in the sports clubs can help teens find their ways adaptively in their future lives (Fraser-Thomas and Côté, 2009; Strachan and Davies, 2015). The social benefits of a sports club membership can contribute to the recursive processes deriving from the broaden-and-build theory of Fredrickson (1998, 2001). Similarly, to the above-mentioned intra-individual benefits, social benefits also require time that can explain why late adolescents benefit most from sports club membership.

Normally, the adolescents’ life becomes more complicated over the teenage years. For example, Britni et al. (2021) found that prevalence of behavioral problems and internalizing disorders, such as anxiety or depression, emerge more frequently in the later years of adolescence. Slobodskaya (2021) found that over the years, adolescents become more aware of their environmental demands. In the later part of adolescence, teens might need more resources to cope with these progressively increasing difficulties. Sports clubs can provide opportunities to build both relevant and useful resources. However, it seems reasonable that utilizing the benefits of the sports club (e.g., positive self-esteem, belonging, and social support) requires time, and when the perceived difficulties peak in late adolescence they should be available.

One of the surprising findings was not observing an interaction of age and sports club membership regarding the negative affects. Based on the prior literature (Sabiston et al., 2016; Doré et al., 2019) and based on the broaden-and-build theory, one could expect that over the years of adolescence, teens belonging to sports clubs will report less and less daily negative affects compared to their non-sportive peers. Further research is needed to explore the potential reasons. One of the potential explanations might be that sports club membership can reduce the feelings of sadness, anxiety, or worries during adolescence equally. The beneficial effect of sports club membership can be hardly accumulated over the years as academic and social challenges increase in parallel with the passing years and sport activities can only maintain their affective benefits without any visible accumulation.

### Limitations

This study has some limitations. First, regarding information on sports club practice, we only know whether the adolescents practiced in a sports club or not. No further information was collected on the number of training sessions per week, the level of practice in competition, and the number of years of practice. However, if they belonged to multiple clubs simultaneously, they were requested to indicate the one they spent the most time practicing with. All this information should be incorporated in future studies. In addition, the distribution between year groups was uneven and it was not controlled for in the analysis. Second, regarding every single student, affects were surveyed 30min after a physical fitness assessment that could have possibly—although homogeneously—influence the retrieval and evaluation of respondents’ affects regarding the previous few days. Although our affect measure showed consistent factor structure and great internal consistency. We have to mention that the temporal stability of the positive and negative factors of the affect measure has not been investigated yet. Future work might either use a more commonly utilized measure or it is also possible to validate this measure. It is also possible that future studies might try to use ecological momentary assessment (Trull and Ebner-Priemer, 2013) if they are interested in short term and less pervasive benefits. The relatively low explained variances of the regression models can also be considered as a limitation of this study.

## Conclusion

In sum, it appears that in the light of the present and prior results, the “Build-and-Broaden Theory” of Fredrickson can provide some hints about the reasons why sports club membership contributes to more positive affects in late adolescence than early adolescence. This theory describes the form of positive emotions as to broaden awareness and their function as to build resources. To confirm these results, further studies with longitudinal and experimental methods are required that could appropriately measure the accumulation of potential affective benefits over time. This is not the only limitation of the present work. Alternatively, the use of other more recognized tools measures should also demonstrate the present results. It would be useful to consider the teens’ level of advancement in their sports club. It is also possible that certain sports have more, and others have less intra-individual and inter-individual affective benefits. Future studies could not only distinguish between these effects, but they can also examine teens’ different trajectories of development and the impact of sports club memberships on the affective landscape of various students. In sum, it appears that the present study only found a reasonable interaction effect; however, further studies are required to explain these preliminary results in detail.

## Data Availability Statement

The raw data supporting the conclusions of this article will be made available by the authors, without undue reservation.

## Ethics Statement

The studies involving human participants were reviewed and approved by Institutional Review Board of 00012476–2021-28-05-109. Written informed consent to participate in this study was provided by the participants’ legal guardian/next of kin.

## Author Contributions

CA, AB, AC, JC, HO, and GO contributed to the study design, literature review, data gathering, manuscript writing, data analyses, and interpretation. All authors commented on the draft and contributed to the final version, approved the publication of the manuscript, and agreed to be accountable for all aspects of the work.

## Funding

GO was supported by the Young Researcher STARS grant from Conseil Régional Hauts de France. AB was supported by the CIFRE n°2020/0331 grant from the Association Nationale Recherche Technologie (ANRT).

## Conflict of Interest

The authors declare that the research was conducted in the absence of any commercial or financial relationships that could be construed as a potential conflict of interest.

## Publisher’s Note

All claims expressed in this article are solely those of the authors and do not necessarily represent those of their affiliated organizations, or those of the publisher, the editors and the reviewers. Any product that may be evaluated in this article, or claim that may be made by its manufacturer, is not guaranteed or endorsed by the publisher.

## Footnotes

^1^Eleven-year-old teens were overrepresented (39.92%), while the 17â€‰years old were underrepresented (2.50%), while the other age groups were the following: 10â€‰years old 4.44%, 12â€‰year old 21.43%, 13â€‰year old 9.81%, 14â€‰years old 7.10%, 15â€‰years old 7.77%, 16â€‰years old 4.19%, and 17â€‰years old 2.82%.

^2^Our items are very similar to other well-established instruments, such as the Multiple Affect Adjective Check List (Zuckerman and Lubin, 1965), the Profile of Mood States (McNair et al., 1971), and the PANAS (Watson and Clark, 1988).
